# Recurrent Acute Neurogenic Pulmonary Edema after Uncontrolled Seizures

**DOI:** 10.1155/2018/3483282

**Published:** 2018-08-19

**Authors:** Daniel C. Sacher, Erika J. Yoo

**Affiliations:** ^1^Department of Internal Medicine, Drexel University College of Medicine, Hahnemann University Hospital, Philadelphia, PA, USA; ^2^Division of Pulmonary, Critical Care, and Sleep Medicine, Drexel University College of Medicine, Hahnemann University Hospital, Philadelphia, PA, USA

## Abstract

Acute pulmonary edema following significant injury to the central nervous system is known as neurogenic pulmonary edema (NPE). Commonly seen after significant neurological trauma, NPE has also been described after seizure. While many pathogenic theories have been proposed, the exact mechanism remains unclear. We present a 31-year-old man who developed recurrent acute NPE on two consecutive admissions after experiencing witnessed generalized tonic-clonic (GTC) seizures. Chest radiographs obtained after seizure during both admissions showed bilateral infiltrates which rapidly resolved within 24 hours. He required intubation on each occasion, was placed on lung protective ventilation, and was successfully extubated within 72 hours. There was no identified source of infection, and no cardiac pathology was thought to be contributory.

## 1. Introduction

Neurogenic pulmonary edema (NPE) is characterized by acute onset of pulmonary edema after a significant injury to the central nervous system (CNS). There are many CNS insults that have been identified as being associated with NPE including traumatic brain injury, subarachnoid hemorrhage, spinal cord injury, meningitis, subdural hemorrhage [1], intracranial hemorrhage, and status epilepticus [2]. NPE was first described by W. T. Shanahan in 1908 in a case series of epileptic patients who developed acute pulmonary edema in the postictal period after grand mal seizures [3] and then later by Moutier in 1918 in World War I soldiers who suffered from bullet wounds to the head [4]. The pathophysiology of NPE is not well understood, but it is postulated that autonomic sympathetic discharge from a CNS injury likely affects systemic and pulmonary circulation by influencing pulmonary capillary pressure and permeability, resulting in pulmonary edema [2, 5, 6]. Much of what is known about NPE comes from clinical case reports and series, and theories about pathophysiology are derived from experimental animal studies. We describe the case of a young man with epilepsy who developed recurrent acute NPE after witnessed generalized tonic-clonic (GTC) seizures on two consecutive hospital admissions.

## 2. Case Presentation

**Figure 1:**
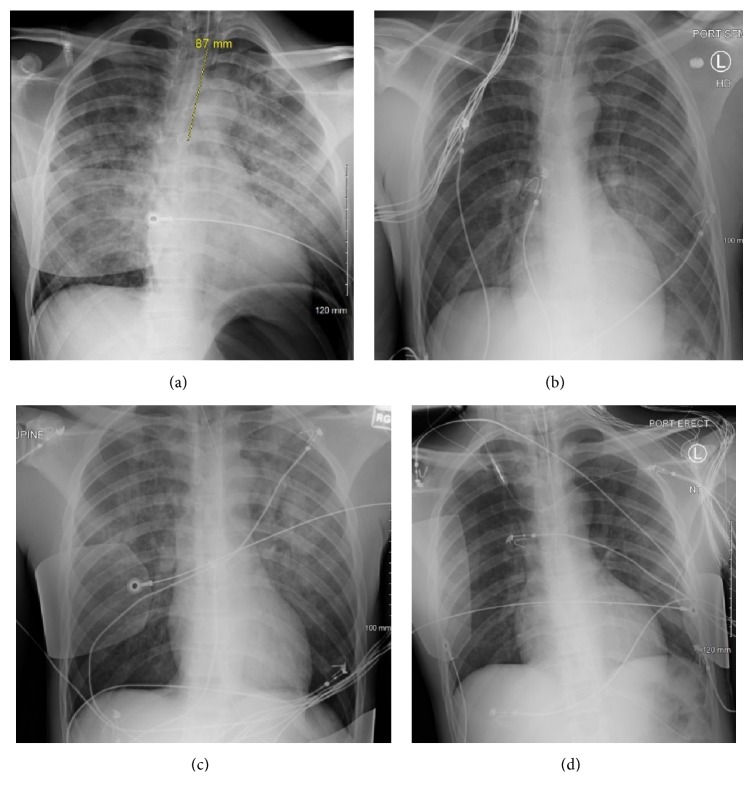
Chest radiographs from each admission. Initial postintubation radiograph (a) from his first admission showing diffuse bilateral infiltrates; repeat radiograph (b) showing resolution approximately 18 hours later. Initial radiograph (c) from the second admission again demonstrating bilateral opacities which resolved (d) within 48 hours.

A 31-year-old man with a history of epilepsy presented twice to the Emergency Room (ER) in the span of 10 days after experiencing GTC seizures. On initial presentation, the patient had a witnessed seizure while at work and was postictal on arrival. While in the ER he again had a witnessed GTC seizure which broke 1 minute after receiving 2 mg of lorazepam intravenously. Almost immediately after the seizure, he became dyspneic and hypoxic despite a Nonrebreather (NRB) mask. He was subsequently intubated for hypoxic respiratory failure. Chest radiograph postintubation showed diffuse bilateral infiltrates (Figure 1(a)), and pink frothy sputum was seen collecting in the endotracheal tube (ETT). The ER team believed that this was blood related to a tongue laceration and suspected aspiration pneumonitis given the radiographic findings consistent with Acute Respiratory Distress Syndrome (ARDS). The patient was loaded with 1 gram of intravenous levetiracetam, per neurology, and then restarted on his home regimen of 1 gram twice daily. He was admitted to the Medical Intensive Care Unit (MICU), started on a course of ampicillin/sulbactam for possible aspiration pneumonia, and, due to a PaO_2_:FiO_2_ of 128 mm Hg, was started on inhaled epoprostenol and placed on lung protective ventilation. Brain computed tomography (CT) showed no acute intracranial abnormality, and continuous electroencephalogram (EEG) showed diffuse background slowing and occasional bifrontal spike and wave discharges that were thought to suggest frontal lobe epilepsy. ENT performed a bedside nasopharyngolaryngoscopy and found no upper airway source of bleeding. Repeat chest radiograph the following day showed rapid resolution of the initial bilateral infiltrates (Figure 1(b)) with PaO_2_:FiO_2_ recovered to >200 mm Hg. His blood, urine, and sputum cultures were unremarkable, and influenza and respiratory syncytial virus polymerase chain reaction were negative. Antibiotics and inhaled epoprostenol were discontinued, and the patient was successfully extubated 48 hours after intubation. MRI brain showed mesial temporal sclerosis primarily affecting the left posterior hippocampus. He was discharged in a stable condition with instructions to take 1.5 grams of levetiracetam twice daily with scheduled neurology follow-up. Upon further history, it was discovered that the patient had been diagnosed with epilepsy one year prior and had a history of nonadherence to his antiepileptic medications.

The patient returned eight days later to the ER after again experiencing two GTC seizures at home. He was similarly postictal and suffered yet another GTC seizure in the ER which lasted for approximately 1 minute and broke after receiving 2 mg of lorazepam intravenously. The patient was subsequently dyspneic and hypoxic. Bilateral diffuse crackles were auscultated, and he was once more intubated for hypoxic respiratory failure. Postintubation chest radiograph showed increased bilateral airspace opacities (Figure 1(c)), similar in appearance to his prior admission. Frothy pink sputum was also seen in the ETT. Due to a difficult intubation, ER staff believed there was again possible oropharyngeal trauma or tongue biting. He was started on antibiotics for a possible pneumonia and admitted to the MICU, and given imaging suspicious for ARDS with a PaO_2_:FiO_2_ of 75 mmHg he was placed on lung protective ventilation. The patient was reloaded on intravenous levetiracetam. Brain CT again showed no acute intracranial abnormalities, and continuous EEG showed diffuse encephalopathy likely secondary to medication effect with no epileptiform activity. Repeat chest radiograph the day after admission showed rapid resolution of bilateral opacities (Figure 1(d)). His cardiac brain natriuretic peptide was within normal limits, there was no growth from blood, urine, and sputum cultures, and a transient postictal leukocytosis resolved. Antibiotics were stopped, and patient was successfully extubated within 72 hours of mechanical ventilation. A transthoracic echocardiogram was performed during this admission that showed a normal ejection fraction, normal diastolic function, no significant valvular disease, and no evidence of pulmonary hypertension.

## 3. Discussion

NPE typically presents in a patient who has a history of epilepsy or neurologic insult and becomes rapidly short of breath within minutes to hours, commonly after a GTC seizure. There is usually tachypnea, tachycardia, and rales or rhonchi on physical exam; hypoxemia and minimal leukocytosis can also occur [7]. Chest radiographs usually show bilateral alveolar opacities without cardiomegaly [8]. There is also a delayed onset form where pulmonary edema takes 12 to 72 hours to appear after a CNS event [9]. Aspiration pneumonia are pneumonitis are common initial diagnoses, but when there are transient respiratory symptoms and radiographic abnormalities that resolve within 24 to 48 hours after seizure, NPE should be strongly suspected [7]. In a review of NPE case reports, authors found that symptom onset occurred less than 4 hours after the neurologic event, more patients were female with a mean age of 31.6 years, one-third of patients had pink frothy sputum, chest radiographs showed bilateral diffuse infiltrates in over 90% of cases, and recovery was very rapid within less than 72 hours in over half of the patients [10].

The diagnosis of NPE is not only a clinical diagnosis, but also a diagnosis of exclusion, usually requiring confirmation of noncardiogenic pulmonary edema after a neurological injury. The most common causes of NPE are epileptic seizures, cerebral hemorrhage, and traumatic brain injury. Diagnostic criteria have been proposed to identify patients with NPE: bilateral infiltrates; PaO_2_:FiO_2_ <200 mm Hg; no evidence of left atrial hypertension; presence of CNS injury severe enough to have caused significantly increased ICP; and absence of other common causes of acute respiratory distress or ARDS, such as aspiration, sepsis, or massive blood transfusion [2]. A differential diagnosis usually includes aspiration pneumonia and cardiogenic pulmonary edema. However, if heart failure can be excluded and if the pulmonary edema is rapid and bilateral without witnessed emesis, then NPE may be considered more likely.

There is much debate in the literature about the pathophysiology of NPE, specifically how neurologic insults influence the pulmonary system. One of the early pathogenic theories called the “blast theory” suggested that increases in the hydrostatic and Starling forces after neurologic injury cause damage to the capillary endothelium, leading to leak of protein-rich edematous fluid in the alveoli [11]. A review of the literature on NPE suggests that permeability and hydrostatic abnormalities likely occur through separate mechanisms. Neuroanatomical sites considered important are the medulla and hypothalamus, as animal models show that lesions to these sites cause surges of sympathetic activity leading to elevated systemic hypertension and increases in left atrial and pulmonary hydrostatic pressure, culminating in pulmonary edema [2, 7, 12]. NPE can also happen without large increases in left atrial and systemic pressures, so a nonhemodynamic mechanism has been proposed from alpha- and beta-adrenergic modulated increases in permeability of the pulmonary capillaries and microvasculature, also related to sympathetic activation [2, 6, 12–14]. Heart failure and left ventricular failure that had been described in some patients are likely due to alpha-adrenergic effects resulting in increased venous return and are exacerbated by norepinephrine effects on pulmonary venous constriction resulting in increased hydrostatic pressures and edema. Treatment of NPE is primarily supportive, including supplemental oxygen, mechanical ventilation if needed, and control of pulmonary vascular pressures [7, 9].

Our patient demonstrated recurrent radiographic bilateral opacities after witnessed GTC seizures on two consecutive admissions. He met the diagnostic criteria proposed with PaO_2_:FiO_2_ <200 mm Hg, bilateral infiltrates, normal echocardiogram findings, a preceding CNS insult, and the absence of suspected sepsis. Although aspiration pneumonia was initially considered in the context of seizures and a difficult intubation, there was no observed emesis, and rapid resolution of infiltrates on repeat chest radiography made the diagnosis of pneumonia less likely. Negative-pressure pulmonary edema (NPPE) was also considered in the differential given the reported difficult intubation. NPPE can be seen in spontaneously breathing patients who have an upper airway obstruction, most commonly infection, tumor, or laryngospasm, leading to very negative intrathoracic pressures causing pulmonary edema and hypoxemia which resolves within 48 hours [15]. The most common cause of NPPE is postextubation laryngospasm following surgery [15], yet our patient developed hypoxia and respiratory failure immediately following GTC seizures. On both admissions he was also noted to have pink frothy sputum in his ETT which has been described in numerous case reports [3, 10], sometimes as severe as hemoptysis [9]. We conclude that acute NPE was the most likely diagnosis in our patient on both occasions, although NPPE cannot be discounted as a possible diagnosis.
